# Chronic Migraine May Be Associated With Postprandial Hypoglycemia in Adult Men: A Case Series

**DOI:** 10.7759/cureus.54987

**Published:** 2024-02-26

**Authors:** Alfred Amendolara, Wyatt D Magoffin, Aparna U Naik, David Sant, John Kriak, Breniman Green, Kyle Bills

**Affiliations:** 1 Federated Department of Biology, New Jersey Institute of Technology, Newark, USA; 2 Department of Biomedical Sciences, Noorda College of Osteopathic Medicine, Provo, USA; 3 Neurology, Migraine and Neurological Rehabilitation Center, Provo, USA

**Keywords:** case series, postprandial hypoglycemia, neurology, hypoglycemia, migraines

## Abstract

Migraine is a common neurological disorder that significantly impacts patients around the world. In the United States, one in six individuals suffers from a migraine disorder. Despite its high prevalence, the etiology of migraine is not well understood. Multiple factors likely contribute to the development of both acute and chronic migraine, making the consensus as to the cause and treatment difficult. Presented here are three case studies involving adult males suffering from chronic migraine. Each subject provided a medical history and underwent physical, psychological, and neurological examinations. In addition, relevant bloodwork and cervical spine X-rays were obtained. Physical examination, laboratory studies, imaging, and psychological metrics were unremarkable with the notable exception of the three-hour oral glucose tolerance tests. All three patients displayed hypoglycemia at three hours. Furthermore, their symptoms markedly improved with the initiation of a ketogenic diet. These data are suggestive of a potential link between postprandial hypoglycemia and chronic migraine. Despite the small sample size, we feel that this report presents possible evidence for a connection between postprandial hypoglycemia and chronic migraine. Furthermore, properly controlled studies of larger sample sizes are required, but we suggest that clinicians consider screening patients for this easily overlooked metabolic disturbance, especially in the absence of other options.

## Introduction

The ongoing Global Burden of Diseases, Injuries, and Risk Factors Study continues to identify migraine as a leading cause of disability worldwide, particularly in individuals younger than 50 years of age [1]. In the United States, the overall prevalence of migraine in men and women is 15.9%, with women having a higher rate at 21% versus 10.7% in men [1-3]. Migraine status has been linked to diet, personal behaviors, metabolic factors, oxidative stress, biochemical functions, and genetics [1,2,4-10]. Furthermore, migraine attack induction has also been associated with a variety of factors, again including diet, behavior, genetics, and biochemical functions [1-3]. Recent studies have provided important new insights into its genetic causes, anatomical and physiological features, metabolic factors, and pharmacological mechanisms [1-9]. However, the critical hodological underpinnings responsible for migraine are not well understood [1-3]. Improved characterization and diagnosis of its clinical features have led to the view of migraine as a complex, variable multisystemic disorder rather than simply a vascular headache. However, the question of how each of these factors, individually or together, contribute to the pathophysiology of chronic migraine remains unanswered.

Chronic migraine is a more severe form of migraine that is defined by the International Classification of Headache Disorders (ICHD) 3rd edition as a headache occurring on ≥15 days per month for more than three months, of which at least eight days per month have features of migraine headache [11]. While chronic migraine is often associated with medication overuse, many other factors may increase the risk for chronicization of migraine headaches [12]. One potentially overlooked factor may be postprandial hypoglycemia, formerly known as reactive hypoglycemia. Postprandial hypoglycemia is a relatively uncommon meal-induced hypoglycemic disorder. The condition is characterized by a pathological drop in blood glucose levels after ingestion of a glucose load. Diagnosis is generally achieved following a fasting three-hour glucose tolerance test although it may be made clinically based on Whipple’s triad (symptoms of hypoglycemia and low plasma glucose and resolution of symptoms upon administration of oral glucose) [13].

In this case series, we present three patients with chronic migraines who showed postprandial hypoglycemia based on a fasting three-hour glucose tolerance test and subsequently responded well to a ketogenic diet.

## Case presentation

Three male patients, two Caucasian and one Hispanic, aged 24, 25, and 25, were seen at the Migraine and Neurological Rehabilitation Center in Provo, Utah, for evaluation of previously diagnosed chronic migraines (Table 1). Following a complete history and physical exam, the complete blood count with differential (CBC), hemoglobin A1c, comprehensive metabolic panel (CMP), and oral glucose tolerance test (OTT) were ordered. Lab analysis was performed by Labcorp (Burlington, North Carolina). All patients had a prior diagnosis of chronic migraine based on the ICHD-3 criteria. All three patients presented with typical migraine symptoms, including severe pulsatile headaches lasting >4 hours, photophobia, and nausea in addition to the specific symptoms listed below. In addition, patients 1 and 2 presented with symptom patterns consistent with possible aura, including reversible numbness and tingling, vision changes, and speech difficulty precipitating headache within one hour. Patient 3 reported non-specific symptoms they considered consistent with possible aura as well. Neuropsychiatric testing was performed using a commercially available test (Creyos, Toronto, Ontario). A psychological performance summary was obtained by a registered nurse for each patient. In addition, all patients received cervical spine X-rays, which were unremarkable. Results of the OTT have been aggregated for further discussion.

**Table 1:** Summary of patient presentation * Approximate number of days qualifying as migraine days based on ICHD-3 criteria. ** Approximate year of prior diagnosis of chronic migraine.

Patient	Age		Race/ethnicity	Headache days per month	Migraine days per month*	Migraine headache characteristics	Aura	Prior treatments	Initial diagnosis**
Patient 1	24	Caucasian		Daily	8-12	Headaches lasting >4 hours, unilateral location, moderate-severe pulsatile pain, associated nausea, and photophobia, triggered by exercise	Yes	Nortriptyline	2020
Patient 2	25	Caucasian		Unavailable	8-12	Headaches lasting >4 hours, unilateral location, moderate-severe pulsatile pain, associated nausea, and photophobia, triggered by exercise	Yes	Caffeine, diphenhydramine	2018
Patient 3	25	Hispanic		Daily	8-12	Headaches lasting >4 hours, unilateral location, moderate-severe pulsatile pain, associated nausea, and photophobia	Yes	Nerve block, botox injection, acupuncture, physical therapy, fremanezumab, unknown oral medications	2015

Patient 1

Initial Presentation

A 24-year-old male presented for treatment of chronic migraine diagnosed in 2020. The patient complained of a constant low-grade headache with migraine-typical escalations characterized by severe, pulsatile pain, photophobia, and nausea occurring eight to 12 times per month and averaging 24 hours in length. He reported the pain spread from the base of his skull. In addition, the patient complained of episodes of numbness and tingling in his right arm, non-specific upper respiratory symptoms, and nausea. These symptoms occurred intermittently but were becoming more common at the time of presentation. It is unclear that the respiratory symptoms were associated with the patient’s migraine headaches although his numbness and tingling precipitated his headaches and are consistent with aura. He stated that concentration and memory had deteriorated recently.

The patient had a past medical history significant for multiple concussions. One concussion occurred during childhood and resulted in loss of consciousness. The second occurred in 2018 after falling while wakeboarding. Of note, the patient’s mother had a history of migraine. At the time of presentation, the patient was taking nortriptyline, which was not providing acceptable relief. The patient reported that jogging and other exercise, schoolwork, and concentration triggered headaches.

Physical Exam and Labs

The patient had an unremarkable CBC, CMP, and hemoglobin A1c. His physical examination was remarkable for slight hyporeflexia, nystagmus, positive left finger-to-nose test, positive heel-to-toe walk with eyes closed, positive Mittelmeyer, increased sensitivity in several dermatomes, and painful temporalis, suboccipital, and shoulder regions. The psychological performance summary was notable only for a below-average score on mental rotations. The results of the OTT were abnormal.

Patient 2

Initial Presentation

A 25-year-old male presented for treatment of chronic migraine, originally diagnosed in 2018. The patient reported experiencing aura consisting of blurry vision, fatigue, and difficulty speaking that progressed to pain at the base of the skull and behind the eyes. He also reported numbness and tingling in the limbs during severe episodes, which precipitated headaches, and occasional light sensitivity. At the time of presentation, his migraine frequency was eight to 12 migraine days per month.

The patient’s past medical history was significant for one concussion with loss of consciousness during childhood, idiopathic hypersomnia, for which he was taking solriamfetol, seasonal allergies for which he had been receiving allergy shots, and a longstanding history of migraines starting at age 16. The patient reported that running often triggered migraines. He also reported that caffeine and diphenhydramine were only occasionally effective at reducing symptoms.

Physical Exam and Labs

At the time of presentation, the patient had an unremarkable physical exam. CBC with differential, CMP, and hemoglobin A1c were also unremarkable, as was his psychological performance summary. The results of the OTT were abnormal.

Patient 3

Initial Presentation

A 25-year-old male presented for treatment of chronic migraines, diagnosed in 2015. In addition to pulsatile headaches, lasting >4 hours, with associated nausea and photophobia, the patient reported burning tight pain at the back of the head and suboccipital region, eye strain, and a baseline headache pain rated at 7/10 on a visual analogue scale (VAS). The patient reported that this pain peaked to 10/10 on a VAS daily. The patient reported that schoolwork was becoming more difficult and that he was having increasing difficulty focusing and processing information.

Prior to the onset of migraines, and at the time of presentation, the patient had no other significant past medical history. He had tried, unsuccessfully, to treat his migraines with nerve blocks, botox, acupuncture, physical therapy, and one fremanezumab injection. The patient reported that oral medications were not helpful. At the time of presentation, his migraine frequency was eight to 12 migraine days per month, in addition to daily non-migraine headaches. Of note, this patient had an unremarkable brain MRI available at the time of intake.

Physical Exam and Labs

At the time of presentation, the patient had an unremarkable physical exam. CBC with differential, CMP, and hemoglobin A1c were also unremarkable, as was his psychological performance summary. The results of the OTT were abnormal.

Diagnostic assessment

**Figure 1:**
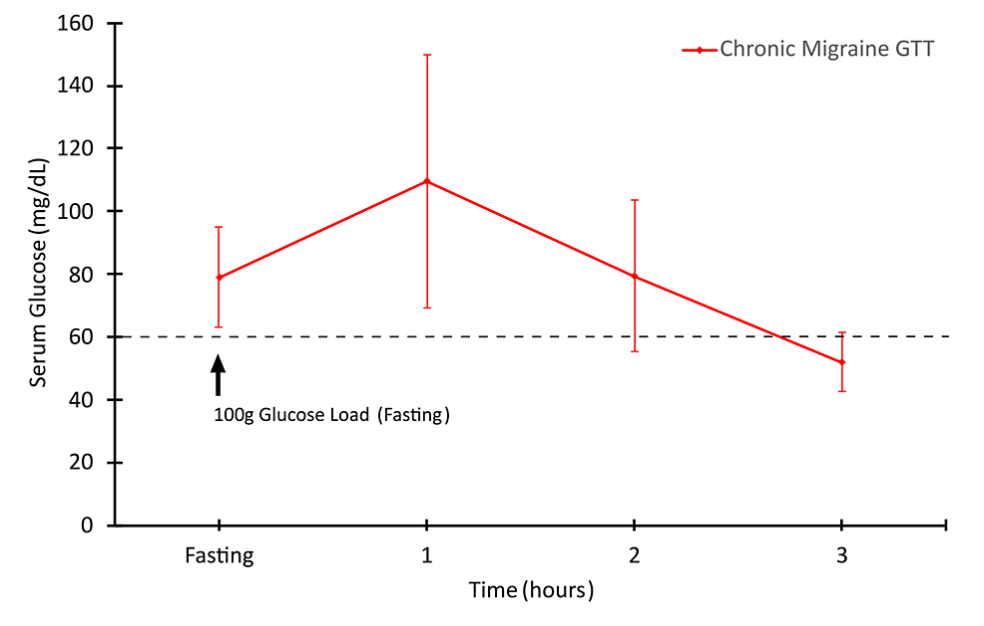
Chronic migraine patients display reduced blood glucose at three hours The results are the mean values displayed with 95% confidence interval. The dashed line indicates the diagnostic cut-off for hypoglycemia.

All three patients presented with chronic migraine, which had been previously diagnosed by outside providers and presented with headaches featuring characteristic migraine symptoms. Other than their self-reported symptoms, physical exams, lab results, and imaging were unremarkable. There were no additional medical diagnoses made as a result of the intake workups, and further in-depth investigation was considered outside of the scope of the current provider. Notably, each patient, during their intake interview, indicated that they experience some level of irritability, fatigue, and other non-specific symptoms concerning for hypoglycemia following meal times. Each patient also associated meal times with the onset of migraine headaches. However, none of these patients had a prior diagnosis of type 2 diabetes or hypoglycemia. This was further confirmed by normal hemoglobin A1c and serum glucose levels (Table 2). These symptoms were concerning for postprandial hypoglycemia. However, the patients had never measured blood glucose after meals. Thus, an OTT was ordered to explore the possibility of this postprandial hypoglycemia. Despite normal hemoglobin A1c values, OTT results at three hours were suggestive of postprandial hypoglycemia (Figure 1, Table 3).

**Table 2:** Hemoglobin A1c and serum glucose values Chart displaying the recorded hemoglobin A1c and serum glucose values of the included patients. SD = standard deviation.

Test	Patient 1	Patient 2	Patient 3	Mean (SD)	Reference range
Hemoglobin A1c (%)	5.6	4.9	5.1	5.2 (0.36)	<5.7
Serum glucose (mg/dL)	70	83	84	79 (7.81)	65-105

**Table 3:** Oral glucose tolerance test results Raw data from patients’ OTT including mean values at each time point. Confidence intervals of the mean were calculated using Student’s T distribution and are written as (lower bound, upper bound). SEM = standard error of mean, CI = confidence interval.

Timepoint	Patient 1	Patient 2	Patient 3	Mean (SEM) n = 3	95% CI
Fasting	70	83	84	79 (3.68)	(63.15, 94.84)
1 hour	124	118	87	109.67 (9.36)	(69.38, 149.9)
2 hours	83	89	66	79.33 (5.62)	(55.13, 103.5)
3 hours	47	56	53	52 (2.12)	(42.70, 61.29)

Therapeutic intervention

After the initial evaluation, these patients were placed on a ketogenic diet consisting of 15 g or less per day of gross carbohydrates. A ketogenic diet was implemented as a non-pharmacological approach to migraine treatment as it has shown promise in recent literature [14]. They were given meal planning support and daily contact via text message and asked to log all food and liquid consumed using the app Carb Manager. They were given ketone urinalysis test strips and asked to test twice per day and send images of test results to verify diet compliance. Throughout the study, all three patients demonstrated consistent adherence to the dietary protocol for the initial three months, followed by a gradual two-month taper period where they increased their carbohydrate intake by 20 g each week. Subsequently, they were instructed to maintain a long-term carbohydrate intake below 180 g per day.

Follow-up and outcomes

Remarkably, within the first three months of adhering to the ketogenic diet, each patient experienced a substantial reduction in migraine frequency, with occurrences of migraine headaches dropping to fewer than four per month in all patients. This improvement was maintained, as evidenced by a follow-up assessment conducted six months after the commencement of the dietary intervention. No adverse effects were reported by any of the patients. However, given that symptoms resolved convincingly, OTT was not repeated at the three- or six-month follow-ups. Hence, it is unclear if there was concurrent resolution of postprandial hypoglycemia as well.

## Discussion

The three cases presented here describe demographically similar patients evaluated for chronic migraine. These patients have no history suggestive of diabetes or other metabolic disorders, yet OTT suggests hypoglycemia. Given the variety of possible triggers of migraine headaches, especially metabolic disturbances, this may present a significant finding [1-3].

Little is known about the link between chronic migraine and postprandial hypoglycemia, a potential metabolic factor in the development and induction of migraine headaches [4,15-19]. Two hypothalamic nuclei are known to play an important role in glucose regulation, the ventromedial and arcuate nucleus. These nuclei contain glucose-excited and glucose-inhibited neurons that function in ways similar to cells found in the pancreas [7]. The ventromedial nucleus has been shown to play a critical role in communicating with peripheral tissues to maintain blood glucose levels within a normal homeostatic range [8,20,21]. In addition, its function is critical in regulating responses to both hypo- and hyperglycemic events through a regulatory role in the secretion of glucagon, glucocorticoids, catecholamines, and insulin [9,22]. Glucose regulation in migraine has long been a contentious topic as reports have often conflicted. These conflicting reports are certainly paradoxical as hypoglycemic symptoms are commonly reported by patients even in the presence of fasting blood glucose levels, HgA1c, and insulin sensitivity that are more characteristic of hyperglycemia. Animal studies suggest that cerebral hypoglycemia elicits an initial glutamatergically-mediated excitotoxic state, leading to mitochondrial dysfunction and oxidative stress characteristic of cortical spreading depression [15]. Subsequently, transient increases in oxidative stress are enhanced as a result of responsive increases in cerebral glucose, which activates nitric oxide synthase and neuronal NADPH oxidase and causes free radical-mediated death of neurons and susceptibility to migraine induction [15].

Despite the existence of a plausible connection between glucose regulation and migraine, there is little existing literature firmly linking postprandial hypoglycemia and migraines [23]. As such, it is difficult to determine from these results whether hypoglycemia is a potential cause of migraines, if migraines can cause hypoglycemia, or if these results are mere coincidence. Although spurious correlation cannot be ruled out, this presents a promising avenue for future investigation.

At present, the authors of this report are conducting a larger trial using continuous glucose monitoring to assess for glucose dysregulation that may be associated with migraine headaches. With a significantly increased sample size and a proper control group, establishing a more robust relationship between glucose dysregulation, including postprandial hypoglycemia, and migraine is possible.

Clinical implications

These results must be interpreted cautiously as this report does not provide evidence of a causal relationship between hypoglycemia and migraine. However, clinicians who regularly interact with migraine patients, especially those with chronic migraines, may benefit from monitoring blood glucose levels, or conducting an OTT at intake. This will provide another data point to use in guiding treatment, and it may provide insight into patients’ individual conditions. In addition, these patients showed improvement when treated with a ketogenic diet, and given the relatively low risk of dietary changes and the relative ease of implementation, we recommend that these dietary modifications be considered.

Further research is warranted to confirm if treating the observed postprandial hypoglycemia significantly improves the patients’ symptoms. We therefore recommend that clinicians explore existing literature and determine if attempting to evaluate for or address postprandial hypoglycemia fits within a reasonable treatment plan. Of note, given the prevalence of comorbidities with migraine, a ketogenic diet may be otherwise beneficial to patients.

Limitations

The primary limitation of this study is the small sample size and lack of control or randomization. Given the nature of case series, strong arguments regarding treatment or causality cannot be made. Notably, the possibility that these three patients coincidentally presented with both postprandial hypoglycemia and migraine cannot be ruled out. Moreover, given the lack of repeat OTT, it cannot be determined if the patients’ postprandial hypoglycemia resolved along with their migraine symptoms. Additional research is currently underway to further examine these findings in a larger cohort of patients.

Furthermore, these patients received their diagnoses elsewhere. Therefore, records of the initial workup were limited. In addition, although the patients indicated that their migraine headaches did not respond adequately to conventional therapy, a complete list of trialed medications and treatments was not available. The patients presented primarily for treatment of these self-described refractory or treatment-resistant headaches; there was limited opportunity to perform a comprehensive multisystem workup to confirm such a diagnosis. Thus, the potential presence of undiagnosed or misdiagnosed, comorbid conditions and an unclear treatment history confounds these cases. Unfortunately, this limits the generalizability of the results presented here, given the wide range of unknowns in each case.

## Conclusions

This case series presents three similar patients, each with postprandial hypoglycemia discovered when evaluated prior to treatment for chronic migraine. Analysis of the collected results showed otherwise healthy adult males with no evidence of diabetes or other metabolic disturbances, but with abnormal OTT results. The patients improved when placed on a ketogenic diet. This reveals a potentially promising correlation between postprandial hypoglycemia and migraine. However, given the limitations of our case series, no firm causality may be established. Future studies will further examine the potential relationship between postprandial hypoglycemia and chronic migraine.
